# Supra-Pubic Puncture of the Bladder

**Published:** 1872-03

**Authors:** A. W. Calhoun

**Affiliations:** Berlin, Prussia


### FOREIGN CORRESPONDENCE.

### SUPRA-PUBIC PUNCTURE OF THE BLADDER.

BY A. W. CALHOUN, M.D.

BERLIN, PRUSSIA, February 25th, 1872.

Perhaps no class of diseases demand more imperatively the immediate attention and interference of the surgeon, and none whose neglect brings more inevitably fatal results than those causing retention of urine. The affection is daily to be met with, and in view of the great importance of early relief, the different modes of treatment should be carefully studied and well understood. It is unnecessary, here, to dwell upon the many causes of this trouble, for they are well known to all—nor upon the more usual methods practiced for its relief, for they are in continual use by those having such cases coming before them; but I desire to say a few words in regard to the treatment by puncture above the pubes, as occasionally called for in the severest forms of retention, and report a successful case thus operated upon by Prof. Langenbeck.

In a majority of the cases where important operations are required for this evil, the patients are old men, with enormously enlarged prostate glands, whose different lobes, gradually increasing, so encroach upon the prostatic portion of the urethra as not only to importantly diminish its calibre, but to alter its form and position. This portion is materially lengthened by the growth of the prostate, and instead of passing through the gland in a normal, slight curve, becomes tortuous, bending in different directions, and forming, as it were, various angles, which abnormal condition continues to increase till all exit to the contents of the bladder is cut off. With the urethra in such a state, it is easily seen what difficulties are to be encountered in the introduction of instruments, of whatever shape, length or material. Extremely seldom, if ever, is an operation upon the bladder necessary from the

*ordinary* strictures of the urethra, as these can always be overcome by the skillful use of proper instruments. It is maintained by some of our best surgeons that operations upon the urethra itself, for the cure of strictures, are unwarrantable, since the passage of the smallest bougies or catheters never fails in the hands of practiced and competent surgeons, and when an entrance has once been made, nothing remains to be done aside from the gradual dilatation. Of this I have nothing here to say. Of course, no reasonable man would puncture either above the pubes or through the rectum, without having previously sought relief for his patient in all other known remedies or means.

The results of the operation are usually unfavorable, from quite a combination of causes, as, for example, the age of the patient, such obstinate retention occurring most usually, if not invariably, in aged persons, with great hypertrophy of the prostate; the length of time transpiring between the first appearance of the absolute stricture and the operation, several days generally intervening, when the whole system has become saturated by the poisonous ingredients of the urine, and the individual already doomed; the frequent severe inflammation, consequent upon the operation, attacking the bladder and adjacent parts, producing cystitis, peritonitis, etc.; the great shock resulting from the severity of the operation and the sudden emptying of the bladder, filled for many hours to its utmost extent. These and still other reasons serve to give a more than doubtful prognosis to nearly all such cases.

Puncture through the rectum is neither so safe or convenient as above the pubes. When the enlargement exists in the lateral lobes as well as in the middle, (which is usually the case), a very large, if not the larger part of the inferior fundus of the bladder, uncovered by the peritonæum, is overlapped, as it were, by the hypertrophied gland, so that a puncture made behind it is very apt to injure the easily-inflamed peritonæum, by the instrument passing through the membrane, or so near as to make probable the extension of the inflammation from the neighboring parts to it.

Again: there is no small amount of trouble in retaining the canula in position, as the contraction of the rectum and the passage of fæces are inclined to throw it out, as has more than once occurred; and when once out, it is a most difficult matter, if not an impossibility, to replace the instrument through the same puncture.

Fortunately, cases occur quite seldom in which it is necessary to resort to this operation, and statistics show most unfavorable results. If I remember correctly, only three such operations were performed in the "University College Hospital," of London, in more than thirty years of its existence, and during the first twenty-one years, only one.

With the bladder filled with urine to great tension, and pushing forward prominently, the operation itself is one of the simplest; and as the organ, gradually filling and rising in the abdominal cavity, carries up at the same time the reflection of peritonæum, there is but little risk of wounding this membrane by the usual puncture—viz., just above the pubes in the linea alba. The puncture through the symphysis, instead of combining the advantages of the higher operation with fewer dangers, as it was intended, is simply a complica-cation without any material benefits; and in such matters, the simpler, the better.

Even one successful case is a strong argument in favor of its practice, when other modes have been sufficiently but fruitlessly tried, and necessity demands immediate aid for the sufferer. Such a case I have now to report.

S. C——, a man aged sixty-eight years, was brought into Clinic, December 8th, 1871, on account of retention of urine. Face of a pale, anxious character, expressive of great pain and extreme debility. Has previously had good health, with no local bodily trouble till five days ago, when he presented himself with retention from enlarged prostate gland, which, with some difficulty, was relieved by the introduction of the catheter by one of the assistants in the Hospital. On rising from bed on the morning of the 7th, he again found himself unable to pass water, although the bladder was tolerably full, containing the accumulation of the previous night. During the 7th, and the following day, repeated efforts were made by private physicians, called in to see him, to enter a catheter, with no other result than that of producing several false passages and intense inflammation along the urethra. After nearly forty-eight hours of absolute retention, he is brought into the Clinic of Prof. Langenbeck, with the above general appearance, and with genital organs immensely swollen from recent rough handling. The introduction of instruments of various sizes and shapes is attempted, while the patient is under the influence of chloroform, but they invariably pass into some of the many false passages. The bladder is distended to its utmost, reaching nearly as high as the umbilicus; and fearing to delay longer, the supra-pubic puncture is decided upon, since examination per rectum showed the prostate too much enlarged to permit puncture, except too far back. The long, slightly-curved trocar is used, and entered in the median line, half-way between the umbilicus and symphysis pubis, and a very large quantity of offensive, bloody urine drawn off. The canula is retained in the wound and bladder by suitable dressings, and the patient ordered strong fluid diet and weak stimulants.

December 9th.—Has rested quietly since the operation, with no fever and but little pain; still passing bloody urine through canula; bowels to be opened every few days by laxatives.

December 16th.—Fever for the last few days, of a low character; no urine passes as yet through the urethra, the prostate still continuing enlarged.

December 26th.—On the 12th day after puncture the catheter was introduced, and the contents of the bladder brought away through the natural channel.

From this time a milk-warm solution of carbolic acid (one part of the acid to one hundred of distilled water) was injected through the catheter once daily, and drawn off after a few moments. Since the first injection, the improvement has been quite rapid in all respects. All fever has disappeared. An ice-filled rubber bladder was for a time applied over the lower part of the abdomen, which had the effect of relieving the pain and fever in the parts. Otherwise the treatment has been of the simplest kind, consisting chiefly of strong diet and injections of the carbolic acid. The prostate but little larger than natural.

January 23d, 1872.—Patient walked into Clinic to-day, having made a perfect recovery. The wound has healed, and the urine passes without any impediment or assistance; to use his own words, "he now has control of his formerly unmanageable bladder."

Since commencing the above, I have had the opportunity of seeing, in the Pathological Institute of Prof. Virchow, the post-mortem of a case of stricture relieved the second or third day, and dying a few hours afterward, and which is a fair specimen of the course taken by such diseases, and a warrant to the performance of any of the severest operations, rather than allow the affection to progress to an unreasonable extent unrelieved. The main stricture was seated in the membranous portion, with slight enlargement of the prostrate. Several false passages existed in the urethra, all of which presented gangrenous appearances. The bladder was quite thick, and, together with the prostate, infiltrated with pus. The various neighboring soft parts in the pelvis were somewhat implicated, showing, at different points, patches and sacks of pus. Hemorrhagic nephritis had developed itself in a high degree, the inflammation having extended along the ureters. All of these changes took place in less than three days.

Considering the age of this patient, and the severity of such troubles and such operations upon the general system, his complete recovery is somewhat surprising; and this operation affords us at least one more chance to save the sufferer when all others have failed, and frees the medical attendant from the mortification of seeing his patient die unalleviated.

